# Clinical outcomes in typhoid fever: adverse impact of infection with nalidixic acid-resistant *Salmonella typhi*

**DOI:** 10.1186/1471-2334-5-37

**Published:** 2005-05-18

**Authors:** Tamilarasu Kadhiravan, Naveet Wig, Arti Kapil, SK Kabra, K Renuka, Anoop Misra

**Affiliations:** 1 Department of Medicine, All India Institute of Medical Sciences, New Delhi, India; 2 Department of Microbiology, All India Institute of Medical Sciences, New Delhi, India; 3 Department of Paediatrics, All India Institute of Medical Sciences, New Delhi, India

## Abstract

**Background:**

Widespread use of fluoroquinolones has resulted in emergence of *Salmonella typhi *strains with decreased susceptibility to fluoroquinolones. These strains are identifiable by their nalidixic acid-resistance. We studied the impact of infection with nalidixic acid-resistant *S. typhi *(NARST) on clinical outcomes in patients with bacteriologically-confirmed typhoid fever.

**Methods:**

Clinical and laboratory features, fever clearance time and complications were prospectively studied in patients with blood culture-proven typhoid fever, treated at a tertiary care hospital in north India, during the period from November 2001 to October 2003. Susceptibility to amoxycillin, co-trimoxazole, chloramphenicol, ciprofloxacin and ceftriaxone were tested by disc diffusion method. Minimum inhibitory concentrations (MIC) of ciprofloxacin and ceftriaxone were determined by E-test method.

**Results:**

During a two-year period, 60 patients (age [mean ± SD]: 15 ± 9 years; males: 40 [67%]) were studied. All isolates were sensitive to ciprofloxacin and ceftriaxone by disc diffusion and MIC breakpoints. However, 11 patients had clinical failure of fluoroquinolone therapy. Infections with NARST isolates (47 [78%]) were significantly associated with longer duration of fever at presentation (median [IQR] 10 [7-15] vs. 4 [3-6] days; P = 0.000), higher frequency of hepatomegaly (57% vs. 15%; P = 0.021), higher levels of aspartate aminotransferase (121 [66–235] vs. 73 [44–119] IU/L; P = 0.033), and increased MIC of ciprofloxacin (0.37 ± 0.21 vs. 0.17 ± 0.14 μg/mL; P = 0.005), as compared to infections with nalidixic acid-susceptible isolates. All 11 patients with complications were infected with NARST isolates. Total duration of illness was significantly longer in patients who developed complications than in patients who did not (22 [14.8–32] vs. 12 [9.3–20.3] days; P = 0.011). Duration of prior antibiotic intake had a strong positive correlation with the duration of fever at presentation (r = 0.61; P = 0.000) as well as the total duration of illness (r = 0.53; P = 0.000).

**Conclusion:**

Typhoid fever caused by NARST infection is associated with poor clinical outcomes, probably due to delay in initiating appropriate antibiotic therapy. Fluoroquinolone breakpoints for *S. typhi *need to be redefined and fluoroquinolones should no longer be used as first-line therapy, if the prevalence of NARST is high.

## Background

Typhoid fever is a common illness in developing countries like India [1] and is a potential threat to developed nations, in an era of increasing air travel and global operations [2]. In the absence of appropriate chemotherapy, typhoid fever was often a fatal illness and introduction of effective antibiotic therapy in 1950s led to a sharp decline in the rates of complications and mortality due to typhoid fever [3]. However, in early 1990s multidrug-resistant strains of *Salmonella enterica *serotype *typhi *(MDR-ST) that were resistant to all the three first-line drugs then in use, namely chloramphenicol, amoxycillin and co-trimoxazole emerged, and sooner MDR-ST became endemic in many areas of Asia, including India [4]. This change in pattern of susceptibility was reflected even in places far away, such as the United Kingdom [5] and the United States of America [6]. Fluoroquinolones are very effective against MDR-ST, achieving fever clearance in less than four days with cure rates exceeding 96%, and are currently the first-line drug for the treatment of typhoid fever [7].

However, towards the end of the last decade, it was observed that fever took longer time than before to clear, and at times surprisingly failed to respond to ciprofloxacin therapy [8-10]. These isolates had comparatively higher minimal inhibitory concentrations (MIC) of fluoroquinolones, although they were susceptible to fluoroquinolones by conventional disc diffusion testing and recommended MIC breakpoints [8-10]. Nevertheless, such strains of *S. typhi *are resistant to nalidixic acid and it was noted that clinical response to fluoroquinolones in patients infected with nalidixic acid-resistant *S. typhi *(NARST) was inferior to the response in those infected with nalidixic acid-sensitive *S. typhi *(NASST) strains [11]. However, it is not clear whether fluoroquinolones can still be used as first-line drug for the treatment of typhoid fever, and if used whether this has any adverse impact on clinical outcomes other than treatment failure such as development of complications and morbidity assessed in terms of total duration of illness. In this scenario, the present study was undertaken to evaluate the impact of infection with NARST on clinical outcomes in patients with typhoid fever.

## Methods

### Study population

This study was conducted at the All India Institute of Medical Sciences (A.I.I.M.S.) hospital, New Delhi, India. This is a tertiary level medical centre located in north India, serving predominantly to low and middle-income groups of the population. All consecutive patients including children with blood culture-proven typhoid fever, admitted to the A.I.I.M.S. hospital during the period November 2001 through October 2003, and those treated as outpatients during this period and were available for at least one follow-up visit, were prospectively included in the study. Patients were evaluated as per a pre-designed instrument, regarding the demographic details, presenting symptoms, physical and laboratory findings and complications. Response to treatment was assessed in terms of fever clearance time.

### Microbiological methods

All isolates from blood culture were identified by standard biochemical tests and confirmed by slide agglutination test using specific *Salmonella *antiserum (Murex Diagnostics Ltd., UK). Antibiotic susceptibility of the isolates was determined by disc diffusion method using 5 μg disc of ciprofloxacin and 30 μg disc of nalidixic acid (HiMedia Laboratories Ltd., India), as per the National Committee for Clinical Laboratory Standards (NCCLS) guidelines and interpretive criteria [12]. The isolates were also tested for susceptibility to chloramphenicol (30 μg), amoxycillin (10 μg), co-trimoxazole (1.25/23.75 μg) and ceftriaxone (30 μg) (HiMedia Laboratories Ltd., India) by disc diffusion method [12]. Minimum inhibitory concentrations (MIC) of ciprofloxacin and ceftriaxone were determined by the E-test method, using commercially available strips (AB Biodisk, Sweden), as per manufacturer's specifications.

### Definitions

A case of typhoid fever was defined as one that presented with a febrile illness, whose blood culture yielded *S. typhi*. Susceptibility break points to various antibiotics tested, were taken as per NCCLS definitions [12]. Anaemia was defined as haemoglobin less than 100 g/L. Leucopenia was defined as total leucocyte count less than 4 × 10^9 ^cells/L and thrombocytopenia as platelet count less than 100 × 10^9^platelets/L. Leucocytosis was defined as total leucocyte count more than 11 × 10^9 ^cells/L. Liver enzymes were considered as elevated if the levels were more than two times the upper limit of normal (cut-off of 100 IU/L for both alanine aminotransferase and aspartate aminotransferase; 560 IU/L for alkaline phosphatase). Fever clearance time was defined as the time from the start of appropriate antibiotic therapy to the first instance, when oral temperature fell to 37.5°C and remained below that level continuously for 48 hours. Clinical failure of fluoroquinolone therapy was defined as continuing fever, even after five days of continuous treatment with a fluoroquinolone.

### Treatment of typhoid fever

The choice of antibiotic therapy was at the discretion of the treating physician. The practice in general, was to prescribe oral ciprofloxacin (adults: 500 mg b.i.d.; children: 15 mg/kg/day) or ofloxacin (adults: 400 mg b.i.d.; children: 15 mg/kg/day) or cefixime (20 mg/kg/day) for outpatient treatment and to admit the patient for in-patient care, if felt by the treating physician to be not responding to therapy or acutely ill. Hospitalised patients received parenteral antibiotic therapy, either ceftriaxone (adults: 2 g b.i.d.; children: 75 mg/kg/day) or a fluoroquinolone or a combination regimen, as decided by the treating physician.

### Statistical analysis

All statistical analyses were done using a software package (SPSS for Windows, Version 10.0.1, SPSS Inc., Chicago, IL). Continuous variables are presented as mean ± standard deviation (SD) or as median with interquartile range (IQR). Categorical variables are expressed as proportions. Independent sample t test or Mann-Whitney U test as appropriate was applied for the comparison of continuous variables between two groups and categorical variables were compared by chi-squared test or Fisher's exact test. Correlation between two continuous variables such as total duration of illness and duration of prior antibiotic intake was tested by Pearson's product moment correlation. Comparison of continuous variables between more than two groups was done by Kruskal-Wallis test. P-value less than 0.05 was considered statistically significant. All tests were two-sided.

## Results

Over a period of two years, 60 patients (hospitalised: 49, outpatients: 11) with blood culture-proven typhoid fever were included in the study. In patients who had relapse, only the first episode was considered in the analysis. Mean age of the patients was 15 ± 9 years (range: 6 months-39 years). Overall, males were predominantly affected (40 patients, 67%). The predilection for male gender was seen in all age groups (in decades), except in infants and preschool children (0–5 years) where the gender distribution was nearly equal (n = 13; 7 males). The frequency of various symptoms and physical findings at presentation is shown in Table 1.

**Table 1:** Clinical features at presentation, in a study of 60 patients with blood culture-proven typhoid fever

**Characteristic**	**Frequency, overall (n = 60)**	**NARST (n = 47)**	**NASST (n = 13)**	**MDR-ST (n = 22)**	**NonMDR-ST (n = 38)**
Age, years *	15 ± 9	13 ± 9 ^†^	23 ± 8 ^†^	14 ± 9	16 ± 10
Gender (male/female)	40/20	32/15	8/5	15/7	25/13
Fever, days ^‡^	8 (4.8–14)	10 (7–15) ^†^	4 (3–6) ^†^	9 (7–14.3)	7 (3.8–14)
Chills	39 (65)	30 (64)	9 (69)	15 (68)	24 (63)
Anorexia	58 (97)	46 (98)	12 (92)	22 (100)	36 (95)
Abdominal pain	23 (38)	18 (38)	5 (38)	10 (45)	13 (34)
Vomiting	33 (55)	27 (57)	6 (46)	14 (64)	19 (50)
Diarrhoea	27 (45)	22 (47)	5 (38)	11 (50)	16 (42)
Constipation	7 (12)	6 (13)	1 (8)	2 (9)	5 (13)
Intestinal bleeding	1 (2)	1 (2)	--	--	1 (3)
Headache	24 (40)	19 (40)	5 (38)	6 (27)	18 (47)
Altered sensorium	3 (5)	3 (6)	--	2 (9)	1 (3)
Cough	21 (35)	18 (38)	3 (23)	10 (45)	11 (29)
Relative bradycardia	6 (10)	4 (9)	2 (15)	1 (5)	5 (13)
Jaundice	3 (5)	3 (6)	--	--	3 (8)
Hepatomegaly ^§^	29 (49)	27 (57) ^∥^	2 (15) ^∥^	11 (50)	18 (47)
Splenomegaly ^§^	30 (51)	25 (53)	5 (38)	14 (64)	16 (42)

NARST = nalidixic acid-resistant *Salmonella typhi*

NASST = nalidixic acid-sensitive *Salmonella typhi*

MDR-ST = multidrug-resistant *Salmonella typhi*

-- no patients in this category

All data presented as number (%) of patients, except when indicated

* data presented as mean ± SD

^† ^P-value < 0.05 for NARST vs. NASST; compared by independent t test or Mann-Whitney U test

^‡ ^duration of fever at presentation; presented as median (IQR)

^§ ^one patient with underlying chronic myeloid leukaemia was not included

^∥ ^P-value < 0.05 for NARST vs. NASST; compared by Fisher's exact test

All 60 isolates were sensitive to ciprofloxacin by disc diffusion testing and the MIC values of ciprofloxacin for all the isolates were within the susceptible range (0.016–1 μg/mL) as per current NCCLS definitions (susceptible if, MIC ≤ 1 μg/mL). Forty seven isolates (78%) were resistant to nalidixic acid (NARST) and the frequency of resistance to other antibiotics namely, chloramphenicol, amoxycillin and co-trimoxazole was 26 (43%), 28 (47%) and 30 (50%) respectively. About a third of isolates were MDR-ST (22 isolates, 37%) and no isolate was resistant to ceftriaxone (MIC range: 0.016–0.064 μg/mL; susceptible if, MIC ≤ 8 μg/mL).

**Figure 1:**
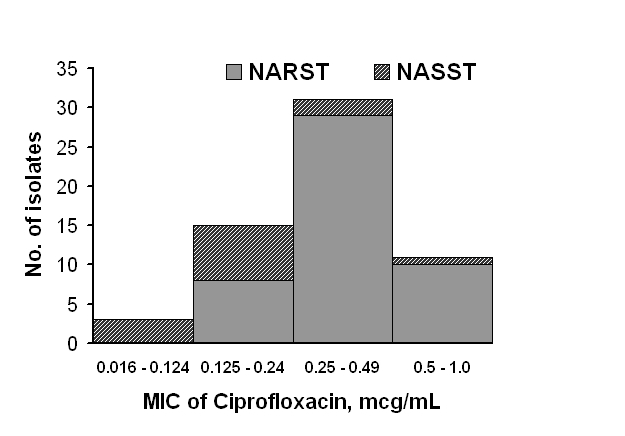
Histogram depicting the distribution of minimum inhibitory concentration (MIC) of ciprofloxacin of 60 *Salmonella typhi *isolates and the proportion of nalidixic acid-resistant (NARST) as well as nalidixic acid-susceptible (NASST) isolates in each column

When compared with NASST, the mean MIC of ciprofloxacin was significantly higher for NARST isolates (Table 2; P = 0.005). However, the distribution of MIC values of ciprofloxacin around the mean, in the two groups was wide and there was considerable overlap. When dichotomised using the median value of MIC (0.25 μg/mL), isolates with MIC values above the median were significantly more likely to be resistant to nalidixic acid (NARST) than were isolates with MIC values below the median (P = 0.013; Figure 1). As a surrogate marker, nalidixic acid-resistance was 82% sensitive and 100% specific for identifying isolates with MIC of ciprofloxacin ≥ 0.125 μg/mL.

**Table 2:** Antibiotic susceptibility and clinical outcomes in a study of 60 patients with blood culture-proven typhoid fever

**Variable**	**All patients (n = 60)**	**NARST (n = 47)**	**NASST (n = 13)**	**MDR-ST (n = 22)**	**NonMDR-ST (n = 38)**
MIC ciprofloxacin, μg/mL	0.33 ± 0.21	0.37 ± 0.21*	0.17 ± 0.14*	0.37 ± 0.26	0.30 ± 0.18
MIC ceftriaxone, μg/mL	0.036 ± 0.016	0.038 ± 0.018	0.031 ± 0.01	0.033 ± 0.017	0.039 ± 0.016
Fever clearance time, days	5.1 ± 3.5	5.5 ± 3.8	3.7 ± 1.3	4.9 ± 2.7	5.3 ± 3.9
Total duration of illness, days	14.3(9.8–21)	16.3(11.4–24)*	9.6(6.8–10)*	14.5(9.3–21.4)	14.3(9.9–22)
*Complications ^†^*	*11 (18)*	*11 (23)*	--	*2 (9)*	*9 (24)*

NARST = nalidixic acid-resistant *Salmonella typhi*

NASST = nalidixic acid-sensitive *Salmonella typhi*

MDR-ST = multidrug-resistant *Salmonella typhi*

MIC = minimum inhibitory concentration

-- no patients in this category

* P-value < 0.05 for NARST vs. NASST; compared by independent t test or Mann-Whitney U test

^† ^includes encephalopathy, hepatitis, lower gastrointestinal bleeding, meningitis and myocarditis. Presented as number (%) of patients; all other data given as mean ± SD or median (IQR)

About half of the patients (47%) presented in the first week of illness. The duration of fever at presentation was significantly longer in patients with NARST isolates (Table 1; P = 0.000) as was the total duration of illness (Table 2; P = 0.000), than in patients with NASST isolates. The frequency of various laboratory abnormalities, according to the susceptibility of the isolate is shown in Table 3. Antibiotic susceptibility pattern had no significant association with any of the physical findings except for hepatomegaly (P = 0.021), which was more frequent in patients with NARST isolates (Table 1). Elevation of hepatic enzymes was a common feature (27 patients, 45%) and in four patients the elevation was marked (> 5 times the upper limit of normal), among which three patients had concomitant hyperbilirubinaemia. Patients with NARST isolates had significantly higher levels of aspartate aminotransferase than patients with NASST isolates (121 [66–235] vs. 73 [44–119] IU/L; P = 0.033). Though the mean levels of alanine aminotransferase and alkaline phosphatase were higher in patients with NARST isolates, these differences were not statistically significant (data not shown).

**Table 3:** Haematological and biochemical findings in a study of 60 patients with blood culture-proven typhoid fever

**Variable**	**Frequency, overall (n = 60)**	**NARST (n = 47)**	**NASST (n = 13)**	**MDR-ST (n = 22)**	**NonMDR-ST (n = 38)**
Anaemia *	19 (32)	15 (32)	4 (31)	6 (27)	13 (34)
Leucopenia *	10 (17)	8 (17)	2 (15)	4 (18)	6 (16)
Leucocytosis *	6 (10)	5 (11)	1 (8)	2 (9)	4 (11)
Thrombocytopenia *	8 (13)	6 (13)	2 (15)	3 (14)	5 (13)
AST > 2 × ULN	27 (45)	23 (49)	4 (31)	9 (41)	18 (47)
AST > 5 × ULN	8 (13)	8 (17)	--	3 (14)	5 (13)
ALT > 2 × ULN	18 (30)	16 (34)	2 (15)	6 (27)	12 (32)
ALT > 5 × ULN	4 (7)	4 (9)	--	1 (5)	3 (8)

NARST = nalidixic acid-resistant *Salmonella typhi*

NASST = nalidixic acid-sensitive *Salmonella typhi*

MDR-ST = multidrug-resistant *Salmonella typhi*

AST = alanine aminotransferase

ALT = aspartate aminotransferase

ULN = upper limit of normal

MIC = minimum inhibitory concentration

-- no patients in this category

* defined by cut-off values as given in the text

All data presented as number (%) of patients

Twenty six patients (43%) had received prior oral antibiotic therapy as outpatients, for a variable duration (2–17 days) before presentation: of which 11 patients (42%) had received fluoroquinolones for at least five days duration and thus were deemed to have clinical failure of fluoroquinolone therapy (others: cefixime – 7 patients [2–15 days]; ciprofloxacin – 4 patients [2–4 days]; amoxycillin – 3 patients [2–4 days]; azithromycin – 1 patient [2 days]). Among these 11 patients, three patients remained febrile even after 10 days of fluoroquinolone therapy. However, all the 11 isolates from patients with fluoroquinolone-failure were sensitive to ciprofloxacin *in vitro*, according to current NCCLS breakpoints (mean MIC: 0.34 ± 0.11 μg/mL; range: 0.125–0.5 μg/mL). Of the 11 instances of clinical failure of fluoroquinolone, in 10 patients the isolates were NARST.

**Figure 2:**
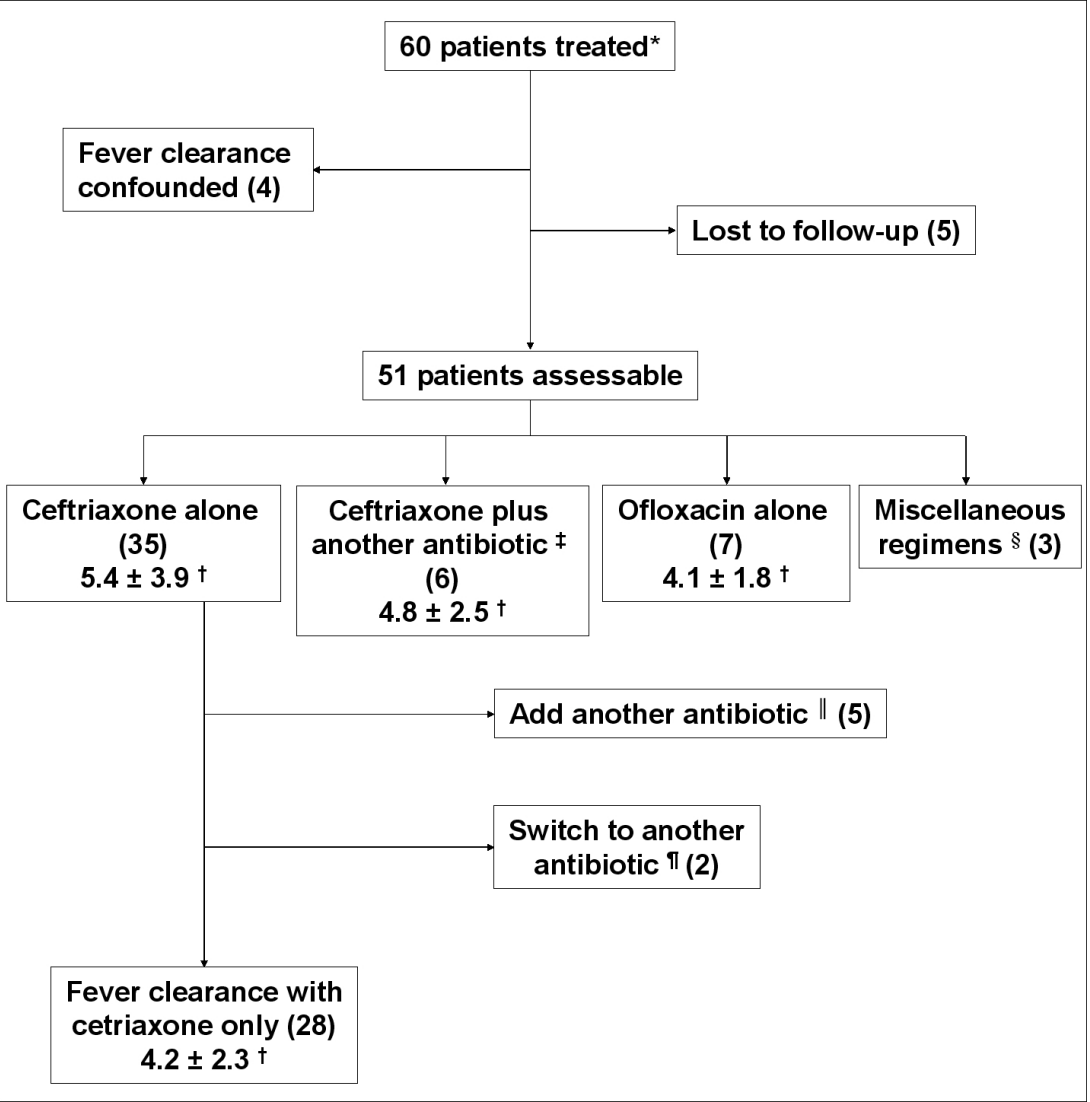
**Antibiotic therapy and fever clearance time in a study of 60 patients with blood culture-proven typhoid fever. **Numbers in parentheses represent the number of patients * 26 patients had received some antibiotic for variable duration before presentation, of which 11 patients remained febrile, even after taking fluoroquinolones for ≥ 5 days ^† ^fever clearance time, presented as mean ± SD (days) ^‡ ^other antibiotics given in combination with ceftriaxone were: ofloxacin (3 patients), amikacin (2 patients) and azithromycin (1 patient) ^§ ^miscellaneous regimens were: cefixime + amoxycillin (1 patient), ceftriaxone + ofloxacin + metronidazole (1 patient) and ceftriaxone + ciprofloxacin + gentamicin (1 patient) ^∥ ^antibiotics added were: amikacin (3 patients) and ofloxacin (2 patients) ^¶ ^switched over to azithromycin (1 patient) and ofloxacin (1 patient)

Fever clearance times were assessable in 51 patients (5 patients were lost to follow-up and in the other 4 patients defervescence was confounded by co-existing conditions). The initial antibiotic regimens were: ceftriaxone alone (35 patients) or in combination with another antibiotic (6 patients) and ofloxacin alone (7 patients) or in combination (3 patients) (Figure 2). Overall, the mean fever clearance time was 5 ± 3.5 days (range: 1–18.5 days). Clearance of fever took seven days or more in 10 patients (20%). Fever clearance time had no significant association with age, gender, antibiotic susceptibility pattern, MIC of ciprofloxacin, MIC of ceftriaxone, or the antibiotic regimen used for treatment. Overall, 11 patients (18%) developed complications, which included encephalopathy (4 patients), meningitis (1 patient), hepatitis characterised by marked elevation of transaminases (4 patients), myocarditis and massive lower gastrointestinal bleeding (1 patient each). All 11 patients who developed complications had NARST isolates. However, this apparent difference between NARST and NASST in the development of complications was not statistically significant (P = 0.1).

**Figure 3:**
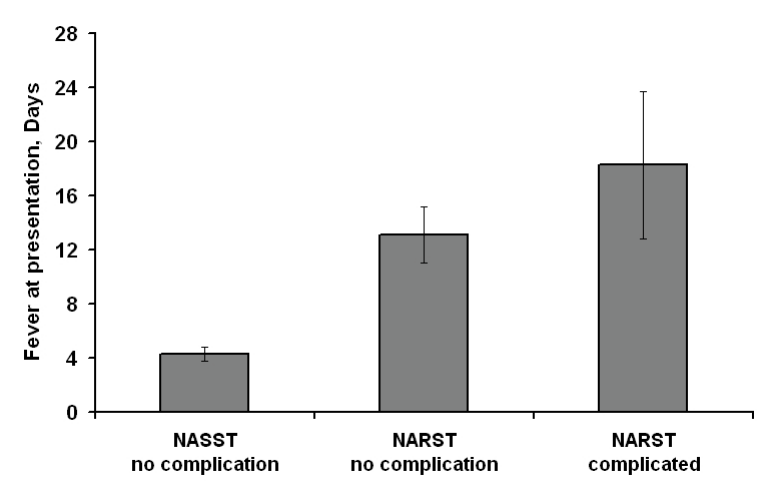
**Duration of fever at presentation, according to nalidixic acid-susceptibility status and presence of complications. **Data presented as mean ± SE NASST = nalidixic acid-sensitive *Salmonella typhi *NARST = nalidixic acid-resistant *Salmonella typhi*

Total duration of illness was significantly longer in patients who developed complications than in patients who did not (22 [14.8–32] vs. 12 [9.3–20.3] days; P = 0.011). This difference became non-significant (P = 0.087) after adjusting for the duration of prior antibiotic intake, suggesting that the difference in the duration of prior antibiotic intake contributed to the apparent difference in the total duration of illness. The duration of fever at presentation was significantly longer in patients who had received prior antibiotic therapy than in patients who had not (12 [7–22.5] vs. 7 [3-10]; P = 0.009) and there was a strong positive correlation between duration of prior antibiotic intake and duration of fever at presentation (r = 0.61; P = 0.000) as well as total duration of illness (r = 0.53; P = 0.000). When patients with NARST isolates were subdivided on the basis of presence or absence of complications, it became apparent that even among patients with NARST isolates, the duration of fever at presentation was comparatively longer in those with complications than those who had NARST isolates but no complications, exhibiting a linear trend (Figure 3; P = 0.001). No significant differences were observed when patients with MDR-ST isolates were compared with the rest, with respect to any of the parameters studied, including rate of complications and total duration of illness (Tables 1, 2 and 3).

## Discussion

The present study brings out the high frequency of nalidixic acid-resistance among *S. typhi *isolates from New Delhi, India. Moreover, this study reconfirms the occurrence of ciprofloxacin-susceptible but nalidixic acid-resistant *S. typhi *isolates and their relation to clinical failure of fluoroquinolone therapy. The frequency of nalidixic acid-resistance as found in the present study (78%) is high in comparison with earlier studies from India, in which it was in the range of 60–67% [13,14]. However, it should be highlighted that these studies were temporally separated, hindering direct comparison between them. Being a hospital-based study, NARST strains might have been inadvertently overrepresented in the study population, since patients infected with NASST are more likely to be successfully treated in the community setting with fluoroquinolones. A community-based study is needed to estimate the actual proportion of infections caused by NARST. An earlier community-based prospective study conducted in New Delhi had found an alarmingly high rate of ciprofloxacin failure (9 out of 63 patients, 14%) as early as 1995–96 [1]. In light of the consistent increase in the proportion of NARST isolates over the last decade, as suggested by data collected at our Institute [15], the high frequency of NARST strains as found in the present study seems to be a true phenomenon rather than due to selection bias. As documented in an earlier study conducted in south India [16], this increase in the frequency of NARST strains is associated with a consistent increase in the MIC levels of ciprofloxacin for *S. typhi *isolates [15].

Interesting is the fact that as per the current NCCLS breakpoints, these NARST isolates with higher MIC of ciprofloxacin would still be classified as being ciprofloxacin-susceptible. Notwithstanding, this subtle increase in the MIC of ciprofloxacin had an adverse impact on the clinical response to fluoroquinolone therapy: in the present study, all patients with clinical failure of fluoroquinolone therapy had isolates with MIC of ciprofloxacin ≤ 1 μg/mL. Hence, it is increasingly being felt that the common fluoroquinolone breakpoints for *Enterobacteriaceae *as set by the NCCLS, are to be reevaluated in the case of *Salmonella *species [13,17,18]. Currently, the NCCLS advises that testing of extraintestinal *Salmonella *isolates for nalidixic acid-resistance may be considered and that fluoroquinolone-susceptible strains of *Salmonella *that test resistant to nalidixic acid may be associated with clinical failure or delayed response in fluoroquinolone-treated patients with extraintestinal salmonellosis [19]. In consonance with earlier studies [13,20], the sensitivity of nalidixic acid-resistance as a surrogate marker for identifying isolates with increased levels of MIC of ciprofloxacin was found to be good in the present study. Thus, testing for nalidixic acid-resistance could be useful as a screening test for further determination of MIC levels of ciprofloxacin.

Since fluoroquinolones exhibit concentration-dependent killing, using higher doses of ciprofloxacin (750 mg b.i.d.) might compensate for this reduced susceptibility [7,21]. Area under the curve (AUC) for serum concentrations attained with this dose is 19.2 ± 1.1 μg.h/mL [22]. This extrapolates to an AUC/MIC ratio of about 77 in the case of half of the isolates encountered in the present study, which is well below the cut-off of > 250 that is required for rapid bactericidal action [23]. Thus using higher doses of ciprofloxacin is also likely to prove ineffective in this population.

Higher frequency of hepatomegaly with greater elevation of hepatic enzymes and a trend for increased incidence of complications in patients with NARST isolates, all suggest that NARST is associated with severe clinical illness. Though the present study did not find a statistically significant association between NARST and the rate of complications, current findings strongly suggest that prior intake of antibiotics not capable of achieving fever clearance is associated with the development of complications. Thus it is reasonable to presume that the use of ciprofloxacin in populations where NARST is widely prevalent would delay the initiation of appropriate antibiotic therapy and thereby would lead to an excess of complications. The current observations are consistent with this hypothesis, though not confirmatory.

Earlier studies had reported that infection caused by MDR-ST was associated with more severe illness than non-MDR-ST infection [24,25]. Noteworthy is the fact that in both these studies antibiotic used as first-line therapy was ampicillin/amoxycillin or chloramphenicol, to which MDR-ST is inherently resistant. Contrary to this, the present study and another study [26] using ciprofloxacin as first-line therapy found no significant differences between MDR-ST and non-MDR-ST infections. This analogy offers ancillary evidence in support of the hypothesis that using fluoroquinolones as first-line therapy in settings where NARST is prevalent, would result in poor outcomes. The other possibility is that development of drug resistance and virulence of the organism might be genetically linked. Blood bacterial counts were reported to be substantially higher in infections caused by MDR-ST than in infections caused by drug-susceptible *S. typhi *[27]. A similar phenomenon is possible in the case of NARST also, accounting for poor outcomes. But the present study was not aimed at evaluating this possibility and this merits further study.

## Conclusion

In this population, nalidixic acid-resistance is very common among isolates of *S. typhi *and is associated with reduced susceptibility to fluoroquinolones *in vitro*. Clinically this translates into frequent failure of fluoroquinolone therapy. However, this is not reflected in current NCCLS breakpoints and hence the fluoroquinolone breakpoints need to be redefined for *S. typhi*. Moreover, use of fluoroquinolones as first-line therapy for typhoid fever delays initiation of appropriate antibiotic in this setting and is likely to result in poor outcomes. For these reasons, fluoroquinolones should no longer be used as the first-line therapy, in populations where nalidixic acid-resistance is common among isolates of *S. typhi*.

## Competing interests

The author(s) declare that they have no competing interests.

## Authors' contributions

TK collected the clinical data, analysed them and drafted the manuscript. NW designed the study, contributed significantly to interpretation of data and revised the draft for important intellectual content. AK participated in the design of the study, coordinated susceptibility testing and revised the draft critically for important intellectual content. SKK participated in the design of the study, contributed significantly to analysis and interpretation of data and revised the draft for important intellectual content. KR carried out microbiological studies and contributed significantly in drafting the article. AM conceived of the study, participated in the design of the study and revised the draft for important intellectual content. All authors read and approved the final manuscript.

## Pre-publication history

The pre-publication history for this paper can be accessed here:


